# Unstable recent intracapsular femoral neck fractures in young adults: Osteosynthesis and primary valgus osteotomy using broad dynamic compression plate

**DOI:** 10.4103/0019-5413.38580

**Published:** 2008

**Authors:** MP Singh, Aditya N Aggarwal, Anil Arora, Ish K Dhammi, Jagjit Singh

**Affiliations:** Department of Orthopedics, University College of Medical Sciences and Guru Teg Bahadur Hospital, Shahdara, Delhi - 110 095, India

**Keywords:** Femoral neck fracture, osteosynthesis, primary valgus osteotomy, broad DCP

## Abstract

**Background:**

Displaced intracapsular femoral neck fractures continue to be a difficult problem to treat. Various treatment modalities and their modifications have been proposed to improve the outcome. Osteosynthesis and primary valgus angulation osteotomy is one of them. Technique and outcome in a consecutive series of recent intracapsular femoral neck fractures in young adults, from a single center, is presented.

**Materials and Methods:**

Fifty-five patients of recent (<3 weeks old) displaced intracapsular fracture neck femur (Garden III and IV, Pauwels III, with or without comminution) in the age group 20-50 years (mean 35.4±10.4 years) were subjected to osteosynthesis and primary valgus intertrochanteric osteotomy using contoured broad dynamic compression plate (DCP). The patients were followed up from two to six years (mean 4.6 years).

**Results:**

Fifty-one fractures united by six months of the index procedure (92.7% union range). Avascular necrosis (AVN) developed in six patients (11%). The other complications were shortening (six), coxa vara (two), infection (two) and delayed union at osteotomy site (one). Excellent results were achieved in 48, good/fair in four and poor in three patients.

**Conclusion:**

Osteosynthesis with cancellous screw and primary valgus intertrochanteric osteotomy stabilized by a contoured broad DCP is a simple, easy to perform, biological treatment. Failure in a particular case can be treated with any appropriate second procedure.

**Level of Evidence:**

IV

## INTRODUCTION

Displaced intracapsular femoral neck fracture in young adults, continues to be a difficult problem to treat. Unfavorable factors like (a) initial vascular insult (resulting in AVN), (b) posterior comminution and (c) vertical fracture line (enhancing chances of nonunion) are largely beyond the surgeon's control.^1^,^2^ A comprehensive classification, treatment protocol, choice of implant and technique, addressing these unfavorable factors influencing the outcome, optimally and predictably are yet to evolve. In an unstable fracture pattern (displaced and/or vertical fracture line and/or comminuted) various treatment modalities and their modifications have been proposed to improve the outcome. Osteosynthesis and primary valgus angulation osteotomy is one of them.^3^–^8^ The indications and the place of this procedure, in the management of adult recent femoral neck fracture is yet not clearly defined. We present outcome of an easy-to-perform, cost-effective technique in a consecutive series of recent intracapsular fracture neck of femur in young adults from a single center.

## MATERIALS AND METHODS

Fifty-five consecutive patients of recent (<3 weeks old) displaced femoral neck fracture in the age group 20-50 years were subjected to osteosynthesis and primary valgus intertrochanteric osteotomy during the period May 1998 to May 2002. These patients were followed up for a minimum period of two years (to look for AVN). Prior approval for this study was obtained from the institutional review committee.

The inclusion criteria were (a) age between 20-50 years (b) traumatic, fresh, closed intracapsular femoral neck fracture of less than three weeks duration (c) Garden Stage III or IV (d) Pauwels Type III fracture line (e) patient able to participate in postoperative rehabilitation program.

The exclusion criteria were (a) pathological fractures (b) patients on oral / injectable steroids (c) clinically detectable major illness like malignancy, chronic renal disease etc. (d) Garden Stage I and II, (e) Pauwels Type I and II, (f) Ipsilateral or contralateral major limb injury affecting treatment and rehabilitation program.

After an informed consent an anteroposterior radiograph of the pelvis with 20° internal rotation at hips was taken for documentation of fracture and comparison with other hip and to calculate the Pauwels angle. Lateral view of the involved hip was taken to assess angulation, displacement of fragments and to note the presence of posterior comminution.

**Figure 1:**
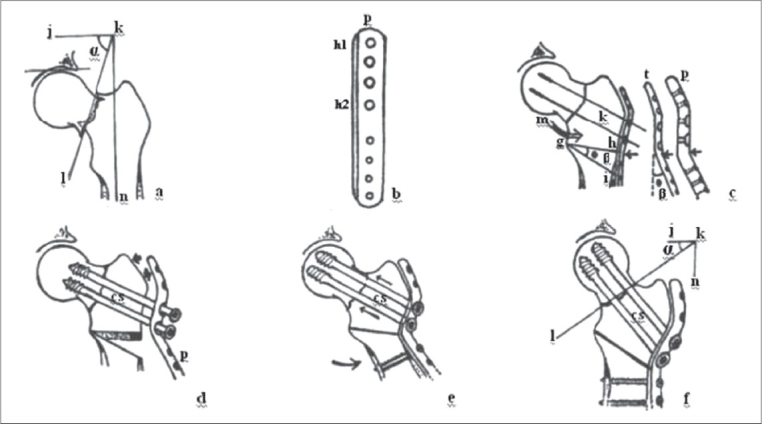
Preoperative drawing, technique and steps of surgery: a) Tracing of preoperative AP radiograph. kn: femoral shaft axis, jk: line perpendicular to femoral axis, lk: fracture line intersecting the perpendicular to femoral shaft axis, α: is the Pauwels angle; b) A broad 8-hole DCP (P) showing h1-h2 widened holes to accommodate cannulated cancellous screws; c) After reduction of fracture, a Hohman's retractor ‘m’ is placed above the lesser trochanter to mark the apex or medial end of osteotomy at the point ‘g’. Template ‘t’ is contoured on the side of the trochanter and proximal femur and then bent further laterally by an angle β- the intended angle of valgisation. The plate (P) is molded, osteotomy is marked as hgi and bent by angle α. Two guide wires κ are passed across the fracture through the plate; d) Cannulated cancellous screws ‘cs’ passed over the guide wire. The wedge ‘hgi’ has been removed; e) The osteotomy is closed by abducting the limb; f) The rest of the plate is fixed to the distal femur using cortical screws. Thus the desired valgisation is obtained and the vertical fracture inclination α becomes more horizontal

The patients were taken up for surgery after pre-anesthetic checkup. Under appropriate anesthesia, the patients were taken on the fracture table for closed reduction (Whitman's Method). Garden's alignment index was used for assessment of adequacy of reduction.^1^ Failing two gentle attempts of closed reduction, an open reduction was performed. The desired length (approximately 12.5 cm) of the lateral cortex of the femur was exposed through lateral/Watson Jones approach. A blunt-tipped Hohman's retractor was passed above the lesser trochanter after palpating it with the finger. This was the guide to the most proximal and medial level of osteotomy. The desired lateral closed wedge osteotomy was marked (intended degree of valgisation 20° to 40° to convert the Pauwels angle to less than 50°). For this procedure a simple modification in an easily available implant was made. The proximal four holes of a standard broad dynamic compression plate (DCP) were enlarged to accommodate 6.5 mm cannulated cancellous screws. The plate is buffed after the screw hole enlargement in the workshop. Templating was done in such a manner that the four holes of the plate were proximal to the proposed osteotomy. The template was bent at the proposed osteotomy site to the desired angle of valgus. The prefabricated broad DCP was contoured according to the template. Two guide wires were passed across the fracture through the proximal holes of the plate under image intensifier control. The desired wedge was removed as the distal limb was abducted to close the osteotomy. Two 6.5 mm cannulated cancellous screws were passed through the plate to stabilize the femoral neck fracture. Distally the femoral shaft was fixed to the plate with 4.5 mm cortical screws [Figure 1].

The acceptability of reduction was graded on the basis of residual angulation and the amount of displacement as excellent (<2 mm displacement and/or less than 5° angulation in any plane), good (2-5 mm displacement and/or 5-10° angulation), fair (>5 to 10 mm of displacement and/or 10-20° angulation)^9^ or poor (>10 mm displacement and/or >20° angulation). Nonunion was defined as persistence or increase in fracture gap at six months, sclerosis of fracture margins, loss of reduction or failure of fixation with implant breakage.

Postoperatively patient was allowed non-weight-bearing two-crutch walking as soon as patient was free from pain. Partial weight-bearing with crutches was permitted at six weeks. Full weight-bearing was allowed after union at the fracture site and the osteotomy site. Routinely, the patients were followed up at three weeks, six weeks, three months, six months and then yearly. The follow-up protocol was modified depending upon the status of union and development of complication, if any.

The outcome was assessed on the basis of hip function and radiographs. The clinical parameters included pain level, range of motion at hip, gait pattern, use of walking aid, stair and chair ability and leg length discrepancy.^10^ Radiological assessment included redisplacement and angulation at fracture site, position and backing out of the hardware, presence or absence of nonunion or avascular necrosis (AVN) and union at osteotomy site.

## RESULTS

There were 35 males and 20 females, age ranging from 20 to 50 years (mean 35.4±10.4 years). The mechanism of injury was road traffic accident in 34 patients and fall from height in 21 patients. Twenty-six fractures (47.3%) were Garden III while 29 fractures (52.7%) were Garden IV. Close reduction of fracture could be achieved in 39 cases (70.9%) while open reduction was resorted to in 16 cases (29.1%). In the latter, peroperatively posterior comminution of varying extent was found in all cases.

**Figure 2:**
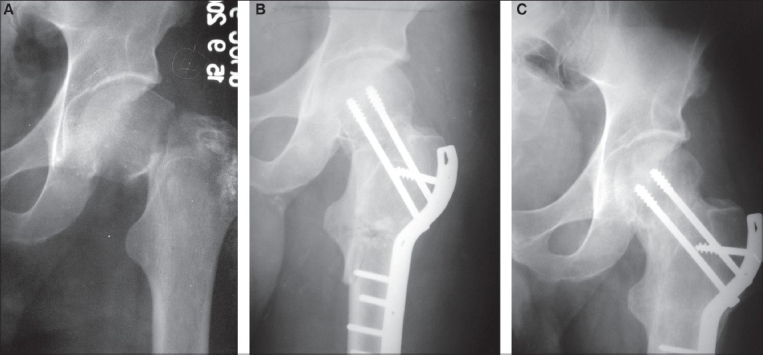
(a): Preoperative X-ray antero posterior view showing Garden III intracapsular femoral neck fracture. (b): Postoperative X-ray antero posterior view showing osteosynthesis of femoral neck fracture with two 6.5 mm partially threaded cancellous screws and primary valgus osteotomy fixed with contoured broad DCP. (c): X-ray ante antero posterior view at two-year follow-up showing sound union. Patient had normal function of hip and excellent results

Excellent reduction could be achieved in 14 patients (25.4%), good in 34 (61.9%) and fair/poor in seven (12.7%). The preoperative mean Pauwels angle of 60±6.4° was converted to a mean of 31.6±5.6° after valgus osteotomy [Figure 2].

**Figure 3:**
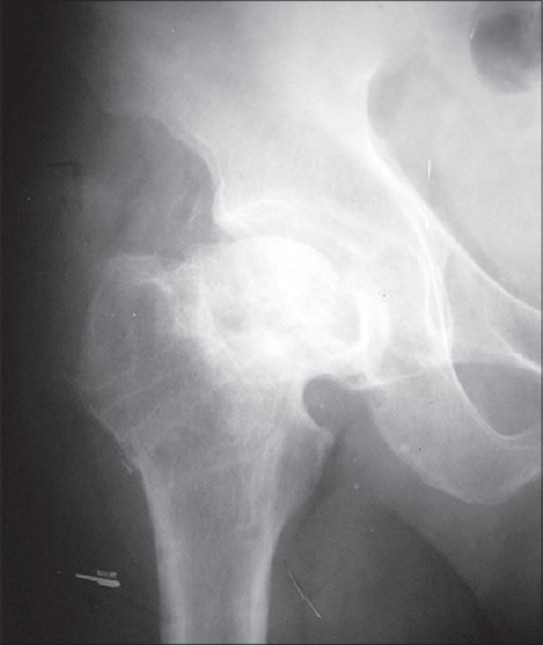
X-ray antero posterior view showing avascular necrosis and collapse of femoral head following primary valgus osteotomy and osteosynthesis, at two years follow-up

The patients were followed up for two to six years (mean 4.6 years). Fifty-one fractures (92.7%) had united by six months postoperatively. Six patients (11%) developed AVN [Figure 3]. In 54 cases osteotomy united by three months. One patient had delayed union at the osteotomy site which took six months to unite. Excellent results were achieved in 48 patients (87.2%), good / fair in four (7.3%) and poor in three (5.5%) by Harris Hip Score.^10^

**Figure 4:**
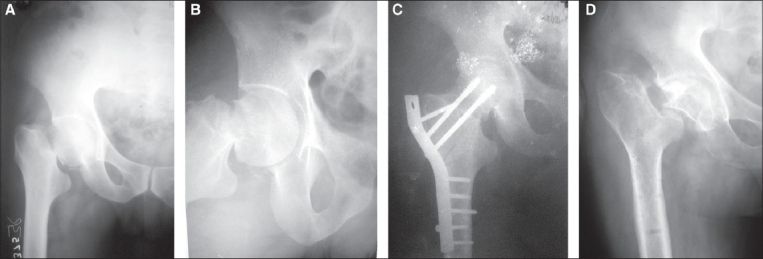
Nonunion following primary valgus osteotomy and osteosynthesis. (a) and (b): Pre-operative antero posterior and Lateral views showing intracapsular fracture neck femur. (c): Postoperative X-ray antero posterior view showing the screws' length is short. This fracture did not unite and the screws cut through the superolateral aspect of the femoral head. (d) X-ray antero posterior view showing un-united femoral neck fracture with healed osteotomy (after 1 year of the index procedure the implants were removed. Patient could walk unaided with painless limp and refused further surgery)

Out of the four patients who developed nonunion [Figure 4], two had excellent/good reduction while two had fair/poor reduction. The correlation between nonunion, AVN and the type of reduction (excellent / good / fair / poor) was not statistically significant (Fisher's Exact test 0.500).

**Figure 5:**
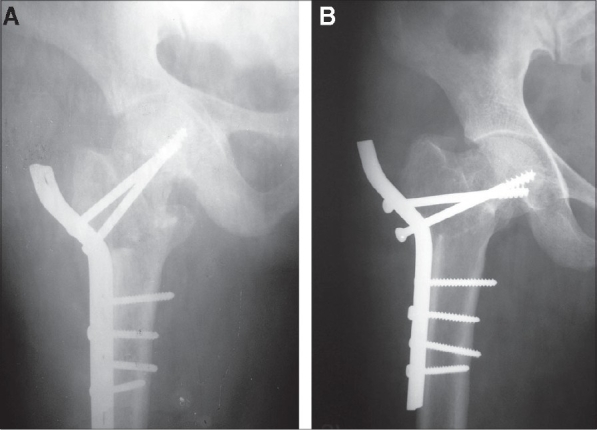
Coxa vara following inappropriate fixation. (a): X-ray antero posterior view showing the plate is not well contoured and standing away from the trochanter. (b): X-ray antero posterior view at four months. The fracture and osteotomy had united but the fracture drifted into coxa vara

Two patients developed isolated coxa vara of more than 110° [Figure 5]. One of these had initial poor reduction and in another, the fracture drifted into coxa vara due to poor fixation.

Two patients developed deep infection. Of these, debridement was done in one patient (this patient developed delayed union at the osteotomy site) and the other patient developed stiff hip (implant removal and girdlestone excision arthroplasty was done in this patient after six months of the index procedure). Shortening of less than 2.5 cm was observed in four and 2.5 to 5 cm in two patients. In no patient lengthening was observed.

The secondary procedures done were debridement, implant removal, girdlestone excision arthroplasty, hemiarthroplasty, muscle pedicle bone grafting and pelvic support osteotomy.

No implant-related complication like disimpaction of screws or breakage of plate occurred in this study.

## DISCUSSION

Displaced intracapsular fracture neck of femur (Garden III and IV), vertical fracture line (Pauwels III) and posterior comminution are associated with increased instability at the fracture site,^2^,^11^ needing evolution in the standard techniques of fixation of femoral neck fractures. The alternatives are (a) modifying internal fixation techniques and implants to enhance fixation,^1^,^12^,^13^ (b) augmenting the posterior defect by muscle pedicle bone graft,^14^ (c) neutralizing unfavorable mechanical forces by doing a valgus osteotomy.^3^–^8^,^15^

Considering the high complication rate following this fracture, as early as 1943, Blount proposed primary osteotomy in recent femoral neck fracture.^4^ The literature on this primary treatment modality is scanty, although this procedure is advocated for nonunion and AVN.^1^ It has not gained widespread acceptability possibly because of (a) invasiveness of the procedure and (b) apprehension of difficulty in conversion total hip replacement (THR) for failed cases. However, Iwase *et al*. did not encounter any major difficulty during conversion THR, done for failed intertrochanteric valgus osteotomy.^16^

Various authors have proposed biomechanical reasons for valgus osteotomy and have shown good results [Table 1]. Bado emphasized better predictability of results.^3^ Thomas King, in 1950, performed internal fixation and primary osteotomy in 38 recent (less than three weeks old) fractures. He hypothesized a possibility of reduction in the incidence of AVN compared to only nailing.^6^ Srivastava *et al*. did osteosynthesis and primary valgus osteotomy in 27 cases of femoral neck fractures and concluded that an intertrochanteric osteotomy gave gratifying results and no salvage procedure was required after primary valgus osteotomy. However, angulation displacement osteotomy (McMurray's osteotomy) was done in these cases.^15^ Rinaldi *et al*. suggested valgus osteotomy for recent fractures which have a high risk of nonunion.^8^ Hermichen *et al*. hypothesized that posterior comminution will not allow intimate contact between fracture surface.^5^ Scheck observed comminution in 70% of patients of Garden III and IV fractures.^2^ Cavaglia *et al*. highlighted the significance of posterior comminution by incorporating it as a factor foretelling instability in their proposed new classification of fracture neck femur.^11^ However, there is no quantification of posterior communition in the literature, making comparison between various series difficult. Meyer *et al*. described the use of posterior placed muscle pedicle bone graft as an alternative treatment modality to augment the posterior bone defect. They reported nonunion and AVN in 5% cases only, but few could match the results.^14^ The serious concern about this method remains the possibility of further damage to the remaining blood supply of the femoral head.

**Table 1:** Various authors have proposed biomechanical reasons for valgus osteotomy and their results

Authors	Number of cases	Proposed biomechanical reasons for advocating primary valgus osteotomy	Results
Bado (1948)^3^	38	Better predictability of results following this procedure	Good
Thomas King (1950)^6^	38	The osteotomy realigned the fracture and transferred the weight-bearing area of the head laterally. He also hypothesized a possibility of reduction in the incidence of AVN	Union 72%, AVN 4%
Rinaldi *et al.* (1984)^8^	25	Suggested osteotomy for recent fractures which have a high risk of nonunion	Union 100%, AVN 8%
Rotolo *et al.* (1989)^18^	26		Very good union 100%, AVN 15%
Fontanesi *et al.* (1991)^19^	24		Excellent 50%, good 20.8%, fair 8.4%, poor 20.8%, AVN 16.6% non-union 8.3%
Hermichen *et al.* (1991)^5^	41	Hypothesized that posterior comminution precludes an intimata contact between the fracture surfaces and prevented the fracture to rest. The osteotomy not only achieves a valgus position of the fracture but also maintains it during the further course of treatment	Nonunion 4%, AVN 22%
Magu *et al.* (2005)^7^	50		Excellent to good 76%, fair 18%, poor 6%, union 94%, AVN 8%
Present series	55		Nonunion 7%, AVN 11%

A large recent consecutive series of patients treated with contemporary methods of internal fixation in displaced intracapsular fracture neck of femur showed nonunion in 9.85% and AVN in 27% patients.^9^ A meta-analysis by Lu-Yau *et al*. showed an occurrence of nonunion in 23-37% of displaced fractures and a cumulative rate of AVN from 11-19%.^17^ In the present series 4/55 (7%) fractures developed nonunion and 6/55 (11%) developed AVN which is quite low as compared to the series where only osteosynthesis has been used. Conversion of Pauwels angle from a preoperative mean of 60° to a postoperative mean of 31° and favorable outcome despite presence of posterior comminution in many cases in our study, further reinforces the observations in the literature that primary valgus osteotomy may be an effective answer to adverse biomechanical factors. However, many authors use Pauwels classification to treat femoral neck delayed and nonunion and not acute fracture.^1^ However, a postreduction type III Pauwels may be predictive of the outcome.^1^ The valgus osteotomy addresses this issue by reducing the postreduction Pauwels angle.

In the past, various implants used for combined osteosynthesis and primary osteotomy were Smith Peterson nail and angled plate or angled blade plate or dynamic hip screw (DHS).^3^,^4^,^6^,^7^,^20^ The application of fixed angle devices requires precision and is technically demanding.^20^ In the present study, the contoured broad DCP was used which has greater maneuverability and ease of application.

The limitation of the present study is that the clinical superiority of this procedure can only be conclusively established by a randomized control trial comparing osteosynthesis and osteosynthesis combined with primary valgus osteotomy. Such a trial is currently undergoing at our hospital. As and when the results of this trial are obtained, they will be made available. The other limitation of the study is that the pre and postoperative neck shaft angle was not calculated. The strength of the present study is the large number of consecutive patients who were treated at a single hospital.

On the basis of our results, we conclude that osteosynthesis with cancellous screw and primary valgus intertrochanteric osteotomy stabilized by a contoured broad DCP is a simple, easy-to-perform biological treatment modality with good functional results in recent displaced vertical intracapsular fracture neck of femur. Failure in a particular case can be treated with any appropriate second procedure.
